# Perenniality, more than genotypes, shapes biological and chemical rhizosphere composition of perennial wheat lines

**DOI:** 10.3389/fpls.2023.1172857

**Published:** 2023-05-08

**Authors:** Marta Bertola, Laura Righetti, Laura Gazza, Andrea Ferrarini, Flavio Fornasier, Martina Cirlini, Veronica Lolli, Gianni Galaverna, Giovanna Visioli

**Affiliations:** ^1^ Department of Food and Drugs, University of Parma, Parma, Italy; ^2^ Wageningen Food Safety Research, Wageningen University and Research, Wageningen, Netherlands; ^3^ Laboratory of Organic Chemistry, Wageningen University, Wageningen, Netherlands; ^4^ Council for Agricultural Research and Economics, Research Centre for Engineering and Agro-Food Processing, Rome, Italy; ^5^ Department of Sustainable Crop Production, Università Cattolica del Sacro Cuore, Piacenza, Italy; ^6^ Council for Agricultural Research and Economics (CREA) Research Centre for Viticulture and Enology, Unit of Gorizia, Gorizia, Italy; ^7^ Department of Chemistry, Life Sciences and Environmental Sustainability, University of Parma, Parma, Italy

**Keywords:** perennial grains, rhizosphere environment, microbial biodiversity, metagenomics, soil metabolomics, soil enzymomics, soil lipidomics

## Abstract

Perennial grains provide various ecosystem services compared to the annual counterparts thanks to their extensive root system and permanent soil cover. However, little is known about the evolution and diversification of perennial grains rhizosphere and its ecological functions over time. In this study, a suite of -OMICSs - metagenomics, enzymomics, metabolomics and lipidomics - was used to compare the rhizosphere environment of four perennial wheat lines at the first and fourth year of growth in comparison with an annual durum wheat cultivar and the parental species *Thinopyrum intermedium*. We hypothesized that wheat perenniality has a greater role in shaping the rhizobiome composition, biomass, diversity, and activity than plant genotypes because perenniality affects the quality and quantity of C input – mainly root exudates – hence modulating the plant-microbes crosstalk. In support of this hypothesis, the continuous supply of sugars in the rhizosphere along the years created a favorable environment for microbial growth which is reflected in a higher microbial biomass and enzymatic activity. Moreover, modification in the rhizosphere metabolome and lipidome over the years led to changes in the microbial community composition favoring the coexistence of more diverse microbial taxa, increasing plant tolerance to biotic and abiotic stresses. Despite the dominance of the perenniality effect, our data underlined that the OK72 line rhizobiome distinguished from the others by the increase in abundance of *Pseudomonas* spp., most of which are known as potential beneficial microorganisms, identifying this line as a suitable candidate for the study and selection of new perennial wheat lines.

## 1 Introduction

Nowadays, intense annual crop production is causing a global exacerbation of natural resources degradation and biodiversity loss (Veerman et al., 2020) which leads to remarkable adverse impacts on essential ecosystem services (Pimentel et al., 2012). In addition, conventional crops are threatened by climate change that strongly impacts agricultural yields (Veerman et al., 2020). Therefore, it is necessary to adopt sustainable agricultural practices that could either decrease the agricultural impacts on climate and at the same time enhance agriculture resilience. The cultivation of perennial grain cropping systems, mostly in marginal land, has been proposed as an innovative method to face climate changes and restore soil health (Glover et al., 2010; Audu et al., 2022b).

The advantages of perennial crops include a longer growing season, a permanent soil cover that doesn’t require intensive soil tillage and seedling, and a deep and dense rooting system (DeHaan et al., 2005; Glover et al., 2010; Pimentel et al., 2012). These features are expected to reduce soil erosion, ensure more efficient use of nutrient and water, also in deep soil layers, and increase soil carbon (C) sequestration, providing fundamental advantages for climate change mitigation and adaptation (DeHaan et al., 2005; Sprunger et al., 2018). Permanent soil cover and reduced soil disturbance also support highly structured and complex food webs, thereby boosting functional biodiversity and conditions for soil diversity conservation (Rasche et al., 2017; Sprunger et al., 2019). Moreover, extensive root network and allocation of belowground carbon, in term of root exudates and root debris, have the potential to promote plant–microbial linkages with important implications for nutrient cycling and ecosystem functioning (Wardle David et al., 2004; Hargreaves and Hofmockel, 2014; Audu et al., 2022b). Indeed, the development of perennial root systems during the plant growth represents a continuous supply of C to soil that stimulates microbial biomass and activity and can shape microbial community composition over time (Culman et al., 2010; Hargreaves et al., 2015; Rasche et al., 2017; Audu et al., 2022a; Audu et al., 2022b).

Previous studies about conservative managed annual cropping system have demonstrated that C addition from organic residues application, use of cover crops, and adoption of conservation tillage, can increase microbial biomass and activity compared to conventional one (Ceja-Navarro et al., 2010; Finney et al., 2017; Ghaley et al., 2018). Likewise, perennial herbaceous cropping systems sustain greater and more complex biological communities (Culman et al., 2010), distinguished by higher microbial biomass (Allison et al., 2005; Culman et al., 2010; Liang et al., 2012), activity (Hargreaves and Hofmockel, 2014) and diversity (Allison et al., 2005; Mao et al., 2013). Moreover, long-term use of these practices can select microbes with different life strategies, resulting from changes in soil structure, increased microhabitat diversity and increased abundance and diversity of available C substrates (Mao et al., 2013; Hargreaves et al., 2015; Schmidt et al., 2018). Furthermore, these practices increase the relative abundance of fungi, mainly due to reduced soil disturbance and hyphal network preservation, which may promote soil organic matter (SOM) stabilization and overall ecosystem functioning (Liang et al., 2012; Finney et al., 2017).

Nevertheless, only few recent studies evaluated shifts of microbial communities under perennial grain crops cultivation (Sprunger et al., 2019; Audu et al., 2022a; Audu et al., 2022b). The authors highlighted that perennial grain often need to be fully established (i.e., > 3 years) before changes in microbial community structure are detectable (Sprunger et al., 2019). On the other hand, other authors showed that perennial wheat cultivation can improve microbial biomass and activity, as measured by enzymes activity and basal respiration, compared with cultivation of annual wheat (Audu et al., 2022a; Audu et al., 2022b).

Current tecnhnologies such as -Omics allow us to decipher the complex dynamics of soil microbial communities and their ecological functions. Indeed, -Omics techniques have recently allowed the characterization of the overall soil microbial genetic and functional diversity through high-throughput analysis of soil DNAs (genomics), RNAs (transcriptomics), proteins (proteomics), enzymes (enzymomics), metabolites (metabolomics) and more recently lipids (lipidomics) (Withers et al., 2020; Bertola et al., 2021). The advent of next generation sequencing (NGS) techniques, has hastened the identification of soil microorganism communities predicting also their potential functions by DNA and RNA shot gun sequencing, while enzymomics, proteomics, and more recently metabolomics, have allowed the investigation of soil microbial biological functions (Vogel et al., 2009; Baldrian, 2014; Withers et al., 2020; Macabuhay et al., 2022). In particular, soil metabolites can reflect interactions between microorganisms and plant roots since they determine microbial food webs, regulate soil chemistry, change microbial gene expression, and act as info‐chemicals to mediate microbe‐to‐microbe interactions (Bais et al., 2006; Hu et al., 2018; Macabuhay et al., 2022). Sugars, amino acids and organic acids dominate the metabolite pool of root-associated soils and mediate plant-microbe and microbe-to-microbe interactions that govern the overall microbial community (Bais et al., 2006; Carvalhais et al., 2011; Canarini et al., 2019). Also, recent evidence revealed an important role of lipids in the cross-talk between roots and rhizosphere microbes (Macabuhay et al., 2022). Lipids are a diverse and ubiquitous group of compounds which play many key biological functions, mainly regulating the plasma membrane cellular processes and signaling mediation. Further, the relative diversity and abundance of lipids in soil have been used to investigate microbial communities’ responses to a range of external stressors, hence providing useful insights to changes in microbial function (Brown et al., 2021).

To date, however, there has been no study that apply high-throughput molecular technologies to unravel the mechanisms that shape soil microbial communities under perennial grain crops. Indeed, there is a gap in understanding the plant-induced modulation of the rhizobiome associated with long-term cultivation of new perennial grain lines.

We hypothesized that wheat perenniality has a predominant role respect to plant genotype in shaping the microbial community composition, biomass, diversity, and activity by affecting the quality and quantity of C input – mainly root exudates – and modulating the plant-microbes crosstalk. Therefore, this study aims to examine the evolution of rhizobiome composition and activity of four perennial wheat lines derived from the cross-breeding between common wheat cultivars and *Thinopyrum* spp. (Gazza et al., 2016; Baronti et al., 2022). These four genotypes have been selected from a wider group of nine wheat x wheatgrass derivatives with a relatively high post-harvest regrowth capacity and for their higher nutritional and technological quality (Gazza et al., 2016; Baronti et al., 2022). Rhizosphere microbial communities were evaluated on the first and fourth year of growth of the perennial wheat lines and compared with the rhizosphere microbial communities of an annual durum wheat cultivar and the parental species *Thinopyrum intermedium*. In particular, we analyzed: i) the variations in biochemical activity of a broad range of hydrolytic enzymes, ii) the 16S rDNA and ITS based bacterial and fungal community composition in the rhizospheres by NGS techniques and iii) the variation in primary metabolites and lipids associated to the root system by un-targeted mass spectrometry-based metabolomics and lipidomics. In addition, to get new insights into rhizobiome dynamics over time, we investigated the relationships between microbial communities’ structure (key microbial phyla) and activity (measured as enzyme activity), and how this is modulated by primary metabolites and lipids.

## 2 Materials and methods

### 2.1 Site description

The field experiment was set up in Central Italy at “Montelibretti” experimental farm station (CREA-IT, Rome) (Lat 42°08’N; Long 12°44’E; 20 m a.s.l.) in the Tiber valley. The area is characterized by a sub-humid Mediterranean climate with annual rainfall of 902 mm and mean air temperature of 14.7°C (historical series 1973–2016). The soil is classified as Arenosol with a silty clay loam soil texture. The experimental field (30 m x 5 m) was placed in a flat and homogeneous area of the experimental farm. Prior to planting, the experimental site had hosted common and durum wheat. Four new perennial wheat genotypes (NPGs) selected by (Gazza et al., 2016) (CPI-147235a, CPI-147280b, 11955, OK7211542, hereafter 235a, 280b, 11955 and OK72, respectively), were sown in November 2017 (year 4) and November 2020 (year 1), while the annual durum wheat cv Ardente and the perennial parental species *Thinopyrum intermedium* (Tpi) were sown respectively in November 2020 (year 1) and November 2010 (year 11). The elementary plot consisted of eight rows, 17 cm apart, sown with 400 germinating kernels/m^2^. Plots were fertilized only the first year at a rate of 150 kg/ha of N (commercial urea fertilizer) applied in three top-dressing: at sowing, at emergence and at tillering phases and no irrigation was used all the years of plant growth. Weeds between plots were mechanically controlled, while those within rows were removed by hand.

### 2.2 Rhizosphere sampling

Rhizosphere samples of perennial wheat genotypes (year 1 and 4), annual durum wheat cultivar Ardente and *Thinopyrum intermedium* were collected in June 2021. Three samples of bare soil (BS) were also collected nearby. Four plants per genotype (235A, 280B, 11955, OK72 and *Triticum durum*) and three plants for the *Thinopyrum intermedium* were excavated from the top 20 cm of soil to collect the rhizosphere with two different methods according to its proximity to roots. Hence, the coarse rhizosphere (RS1) was gently separated from roots, while the thin layer of the rhizosphere, closer to roots (rhizoplane) (RS2), was separated by using an appropriate buffer as described in (McPherson et al., 2018).

### 2.3 16S rDNA and ITS -based community analysis

DNA was extracted from BS and RS2 samples starting from 200 mg of soil using the NucleoSpin Soil Kit (Machery – Nagel) according with manufacturer’s protocol and visualized by electrophoresis on 1% (w/v) agarose gels to test for DNA integrity and quantified with Nanodrop ND1000 (Thermo Fisher Scientific, Waltham; MA, USA). The DNA samples obtained were sent to a commercial provider (IGA-Technology, Udine; http://www.igatechnology.com/) for the 16S rRNA and ITS gene amplification and sequencing. Libraries were prepared by following Illumina 16S Metagenomic Sequencing Library Preparation protocol in two amplification steps: an initial PCR amplification using locus-specific PCR primers (16S-341F 5’- CCTACGGGNBGCASCAG -3’ and 16S-805R 5’- GACTACNVGGGTATCTAATCC -3’ for bacteria; ITS1F 5’-TCCGTAGGTGAACCTGCGG -3’ and ITS4R 5’- TCCTCCGCTTATTGATATGC -3’ for fungi) and a subsequent amplification that integrates relevant flow-cell binding domains and unique indices (NexteraXT Index Kit, FC‐131‐1001/FC‐131‐1002). Libraries were sequenced on a MiSeq instrument (Illumina) in paired end 300-bp mode read length and reads were de-multiplexed based on Illumina indexing system. Taxonomic assignment was done using the software Quantitative Insights into Microbial Ecology (QIIME) (Kuczynski et al., 2012). Raw sequences were processed using the QIIME pipelines and the USEARCH algorithm (version 8.1.1756, 32-bit) was applied for chimera filtering, grouping of replicate sequences, sorting sequences per decreasing abundance and OTU identification. The Operational Taxonomic Unit (OTU) were identified by adopting an “open-reference” algorithm where OTUs were built *de novo* with a clustering threshold set at 97%, with sequences that passed a pre-filter step for minimum identity of 90% with any sequence present in the reference database. OTUs in “open-reference” analysis were generated with a minimum of 2 sequenced fragments. The RDP classifier and Reference databases (GreenGene database (version 2013_8) for 16S rRNA gene and UNITEdatabase v.7.2 -UNITE community, 2017- for ITS gene) were used to assign taxonomy with a minimum confidence threshold of 0.50. For bacteria a total of 6,804,064 16S rDNA raw reads were generated, while for fungi a total of 6,698,310 ITS raw reads were sequenced, with an average number of reads per samples of 154,638 and 152,234 for bacteria and fungi respectively. After denoising and filtering they were reduced to 2,790,088 and 2,377,941 respectively (**Supplementary Datafiles S8, S9**). All raw sequences have been uploaded to NCBI under Bioprojects PRJNA826315.

Alpha and beta diversity were both calculated on the total OTU matrices (see **Supplementary Datasets S3, S4**). To compare bacterial and fungal alpha diversity, Shannon Diversity and Simpson indices were estimated with the R vegan package. All indices were analyzed using a two-way ANOVA on generalized least squares model and means were compared with an adjusted Tukey’s pairwise means comparison procedure using emmeans and multcomp packages in R. The beta diversity was evaluated through distance-based redundancy analysis (dbRDA) as described in (Ferrarini et al., 2021). Briefly, differences in OTU patterns among treatments were evaluated *via* a 2-way model (year x line) based on distance-based redundancy analysis (dbRDA). Contrasts between treatments analysed *via* one-way permutational multivariate analysis of variance using distance matrices (ADONIS) based on the Bray-Curtis matrix. Differences in relative abundances of the most representative genus (relative abundance > 0.3% for bacteria and > 0.03% for fungi) between samples were tested by two-way Analysis of Variance (ANOVA). All graphing was performed with the package ggplot2 in R.

### 2.4 Soil enzymes activities and microbial biomass

Enzyme activity analysis (EAA) was performed on rhizosphere (RS1) samples. The assay based on the procedure of (Cowie et al., 2013) tested 17 hydrolytic enzymes involved in the principal nutrient cycles, namely: arylsulfatase (aryS), α-glucosidase (alfaG), β-glucosidase (betaG), α-galactosidase (alfaGAL), β-galactosidase (betaGAL), arabinase (arabin), β-1,4-xylanase (xilo), β-D-glucuronidase (uroni), chitinase/N-acetyl-β-D-glucosaminidase (chit), tripsin-like protease (trip), leucine amino-peptidase (leu), acid- (acP) and alkaline- (alkP) phosphomonoesterase, phosphodiesterase (bisP), pyrophosphate-phosphodiesterase (piroP), inositol-P phosphatase (inositP) and butyric esterase (butir). All enzymatic activities (EA) were measured in triplicate using a heteromolecular exchange procedure (Cowie et al., 2013) and bead-beating to disrupt soil aggregates and microbial cells. Briefly, 0.4 g fresh weight of soil was transferred to 2 mL microcentrifuge tubes together with 1.4 mL of 3% lysozyme containing 0.4 mL of 100 μm glass beads and 0.4 mL of 800 μm ceramic beads. Bead-beating was carried out using a Retsch 400 mill at 30 strokes s−1 for 3 min, followed by centrifugation at 20,000g for 5 min at 10°C. The supernatant containing desorbed enzymes was dispensed into 384-well white microplates containing the appropriate buffers (aryS, acP, inositP, alfaG, betaG, alfaGAL, betaGAL, arabin, xilo, uroni, and chit in morpholine heptane sulfonic acid 100 mM, pH 6; trip, leu, bisP, butir and piroP, in Tri-hydroxymethyl aminomethane 100 mM, pH 7.5; alkP in Tri-hydroxymethyl-aminomethane 100 mM, pH 9.0) to determine the enzymatic activities by fluorometry using 4- methyl-umbelliferyl (MUF) and 4-amido-7 methyl-coumarine (AMC) fluorogenic substrates, with readings taken using a Synergy HT microplate reader (Bio-Tek, Winooski, Vermont, United States). All measurements were taken in triplicate and the activities were expressed as nanomoles of MUF (or AMC) min/g dry soil. Microbial biomass was determined using the double-stranded DNA (dsDNA) content as a proxy (Bragato et al., 2016). DNA was extracted as described by (Fornasier et al., 2014). Briefly, the bead-beating as well as the centrifugation procedure was the same as for the enzyme described above, but the extraction buffer was 0.12 M sodium phosphate, pH 7.8. After diluting the supernatant, the dsDNA was quantified using PicoGreen (Thermo Fisher Scientific, Milan, Italy).

Differences in EA patterns among treatments were evaluated by distance-based redundancy analysis (dbRDA) using the R:vegan package. Four steps were applied: (1) a Bray–Curtis dissimilarity (nonlinear) matrix was calculated on square root transformed data; (2) a principal coordinate analysis (PCoA) was calculated based on the distance matrix, from which the eigenvalues (obtained in the PCoA) were applied to a RDA with 999 permutations to obtain dbRDA axis coordinates for treatments to be plotted as multivariate centroids surrounded by 95% confidence interval ellipsoids; (3) one-way permutational multivariate analysis of variance using distance matrices (ADONIS) based on the Bray-Curtis matrix was conducted for all 999 permutations on all pairwise contrasts tested for differences among treatments; (4) a similarity percentage (SIMPER) was used toidentify among all the enzymes measured the ones that contributes cumulatively less than 90% to dissimilarity in all contrasts. The following cut-off criterion was applied to allow the identification of the enzymes to use in the dbRDA: 90% cumulative contribution to dissimilarity in at least 70% of total number of contrasts.

Analysis of Variance (ANOVA) was performed on dsDNA data and means were compared with an adjusted Tukey’s pairwise means comparison procedure using emmeans and multcomp packages in R.

### 2.5 Rhizosphere metabolomics and lipidomics

#### 2.5.1 Sample extraction and mass spectrometry detection

Rhizosphere primary metabolites and lipids were extracted from RS1. Briefly, 0.5 g of lyophilized samples were aliquoted into 15-ml Falcon tubes together with 2 ml of 60% (vol/vol) methanol in Nanopure water. Samples were vortexed at maximum speed for 15 min at 4°C and subsequently 3.5 ml of ice-cold chloroform were added. Each sample was sonicated for 5 minute and allowed to cool on ice for 1 min. This latest step was carried out for 5 more times. The samples were allowed to completely cool at -80°C for 10 min, then centrifuged at 4500 g for 10 min at 4°C to separate the aqueous and the organic phase. For each sample 1 ml of the upper aqueous phase was dried down completely in a vacuum concentrator (Labconco, Kansas City, MO), and stored at -20°C until chemical derivatization before GC-MS analysis. In turn,1 ml of organic phase was dried down and reconstructed in 1 ml of ternary mix of acetonitrile/isopropanol/methanol before LC-HRMS untargeted lipidomics analysis.

#### 2.5.2 Metabolomics: sample derivatization for GC/MS and data acquisition

The determination of simple sugars, polyalcohols, amino acids and organic acids of rhizosphere soil, was performed following the sample derivatization protocol described by (Swenson et al., 2015), with minor modifications (SI Appendix, Methods). Briefly, each dried sample residue was added to a mixture of 780 µl of dimetilformammide (DMF), containing 0.050 mg of internal standard phenyl-β-D-glucoside, and 20 µl of a solution containing 2 mg/mL of internal standard tetracosane (C24) in hexane. Then, each solution was added to 200 µl of N,O-bis(trimethylsilyl)trifluoroacetamide (BSTFA) with 1% of trimethylchlorosilane, shaken and incubated at 37°C for 30 min. In this way, all the analytes of interest were transformed in their corresponding trimethyl-silyl-ethers.

Aqueous standard solutions containing simple sugars, organic acids and amino acids were used for metabolite identification, based on comparison of analyte typical retention times and mass spectra. Specifically, for sugars and organic acids, solutions at a concentration of 1 mg/mL of each compound were prepared considering: arabinose, fructose, fucose, galactose, glucose, maltose, mannose, melibiose, myo-inositol, palatinose, ramnose, ribose, sorbitol, trehalose, and xylose (Sigma Aldrich) for simple sugars and polyols, and caffeic acid, cinnamic acid cumaric acid, galacturonic acid, gallic acid, glycolic acid, ferulic acid, lactic acid, malic acid, malonic acid, and sinapic acid (Sigma Aldrich) as organic acids. Concerning amino acids, a 1.25 mM solution was obtained by mixing (in a 1:1 ratio) a 2.5 mM Amino Acid Standard H solution (Thermo Fisher Scientific) and a 2.5 mM mixture of remaining amino acids (nor-leucine, tryptophan, asparagine, and glutamine, from Sigma Aldrich) to a final volume of 1 mL. From each standard mixtures, 100 µl were dried, and dissolved in a solution of 800 µl DMF and 200 µl BSTFA and incubated at 60°C for 1h for derivatization.

For each GC-MS analysis, derivatized samples (1 µl) were split injected (1:20 split ratio) into a Thermo Scientific Trace 1300 gas-chromatograph coupled to a Thermo Scientific ISQ mass spectrometer equipped with electronic impact (EI) source. The separation of analytes was achieved using a BP5MS (30 m × 0.25 mm × 0.25 μm, SGE Analytical Science, Milan, Italy) capillary column and helium as carrier gas. Injector and detector temperatures were kept at 280°C. The oven temperature was programmed from 60°C to 280°C at a 15°C/min thermal gradient, as follows: from 60°C for 0.2 min after injection, to 80°C at 15°C/min, hold for 0.2 min, to 280°C at 15°C/min and hold for 15 min, with a total run time of 30 min. Acquisition was performed in the full scan mode with a 40–500 m/z range. The GC-MS raw data files were collected with Xcalibur 2.2 SP1 w Foundation 2.0 SP1. Compound identification was based on comparison of retention times and mass spectra with those of pure standards or spectral information provided by NIST 14 GC-MS library (**Supplementary Dataset S6**). In addition, the derivatization mode applied lead to the detection of different isomers for some metabolites, especially in case of sugars, so both the obtained signals were reported and identified as different position isomers, when possible (**Supplementary Dataset S6**).

The quantification of each identified gas-chromatographic signal was performed by manually integrating its peak area and calculating with respect to the peak area of the selected internal standard (C24 for organic acids and polyalcohols, nor-leucine for amino acids, and phenyl-beta-D-glucoside for sugars). Finally, values were reported as relative percentage on total metabolites.

#### 2.5.3 Lipidomics: UHPLC−TWIMS−QTOF analysis and data processing

The untargeted lipidomics workflow was performed as described previously (Pedrazzani et al., 2021) (**SI Appendix, Methods**). An ACQUITY I-Class UPLC separation system coupled to a Vion IMS QTOF mass spectrometer (Waters, Wilmslow, UK) equipped with an electrospray ionization (ESI) interface was employed for soil lipidomics. Samples were injected (2 μL) and chromatographically separated using a reversed-phase C18 BEH ACQUITY column (2.1 × 100 mm, 1.7 μm particle size) (Waters, Milford, MA, USA). Gradient elution was performed as previously reported (Pedrazzani et al., 2021) by using 5 mM ammonium formate in Milli-Q water/methanol (95:5, v/v) (solvent A) and 5 mM ammonium formate in isopropanol/methanol/Milli-Q water (65:30:5, v/v) (solvent B) both acidified with 0.1% formic acid. The following multistep elution gradient was used: 0.0 min (10% solvent B; 0.40 mL/min) to 1.0 min (50% solvent B; 0.40 mL/min), subsequently 1−5 min (80% solvent B; 0.40 mL/min), and 11.0 min (100% solvent B; 0.50 mL/min). After a 4.5 min isocratic step, the system was re-equilibrated to initial conditions for 2.5 min (10% solvent B; 0.4 mL/min). Samples were permanently kept at 10°C. Mass spectrometry data were collected in positive and negative electrospray mode over the mass range of m/z 100−1200. Source settings were maintained using a capillary voltage of 2.5 kV, a source temperature of 120°C, a desolvation temperature of 500°C, and a desolvation gas flow of 1000 L/h. A TOF analyzer was operated in sensitivity mode, and data were acquired using HDMSE, which is a data-independent approach (DIA) coupled with ion mobility. The optimized ion mobility settings included a nitrogen flow rate of 90 mL/min (3.2 mbar), a wave velocity of 650 m/s, and a wave height of 40 V. The device within the Vion was calibrated using a Major Mix IMS calibration kit (Waters, Wilmslow, UK) to allow for CCS values to be determined in nitrogen. The calibration covered the CCS range from 130 to 306 Å2. The TOF was also calibrated prior to data acquisition and covered the mass range from m/z 151 to 1013. TOF and CCS calibrations were performed for both positive- and negative-ion mode. Data acquisition was conducted using UNIFI 1.8 (Waters, Wilmslow, UK).

Data processing and compound identification were conducted using Progenesis QI Informatics (Nonlinear Dynamics, Newcastle, UK). Each UHPLC-MS run was imported as an ion-intensity map, including m/z (m/z range 100–1200) and retention time, that were then aligned in the retention-time direction (0–15 min). Isotope and adduct deconvolution were applied, to reduce the number of features detected. Features identification was performed against publicly available database including LIPID MAPS, Human Metabolome database (HMDB), and METLIN, as well as by fragmentation patterns, retention times, and CCS. CCS values were searched against “MetCCS Predictor” database containing m/z and CCS values by selecting a ΔCCS of 5% for metabolite matching (Zhou et al., 2016). Based on the Metabolomics Standards Initiative (Summer et al., 2007), metabolites were annotated as level III (putatively characterized), level II (putatively identified compounds), and level I (identified compound). A mix of monoacylglycerol, diacylglycerols and triacylglycerols was run at the beginning, in the middle and at the end of the sample list to monitor system retention time, CCS and mass error stability.

#### 2.5.4 Metabolomics and lipidomics multivariate modeling

Both metabolomics and lipidomics data matrices were independently subjected to unsupervised principal components analysis (PCA) with pareto scaling was performed to check the quality of the raw data. Afterwards, supervised models, including partial least-squares discriminant analysis (PLS-DA) were built and validated using SIMCA software (v. 16.0.2, Sartorius Stedim Data Analytics, Sweden). The variable influence in projection analysis (VIP) was further used to identify the compounds that had the highest discrimination potential (VIP value threshold > 1.2). Moreover, agglomerative hierarchical clustering analysis was applied to metabolite concentration data and soil samples. Similarity was determined by Euclidean distance for analysis of the differences in metabolite concentrations, and clustering was performed using Ward’s linkage. The dendrograms were combined with a heatmap, generated based on z-scores of metabolite concentrations using the MetaboAnalyst platform (Chong and Xia, 2020).

The redundancy analysis (RDA) was employed to identify the relationship between bacterial and fungal community composition and metabolites and lipid accumulation in the perennial wheat rhizosphere. Data correlation between the composition of microbial communities, metabolites and lipids in the rhizosphere was evaluated by Spearman correlation coefficient (p < 0.005) (see **Supplementary Dataset S10**).

## 3 Results

### 3.1 DNA-based bacterial and fungal community composition and diversity

**Figure 1:**
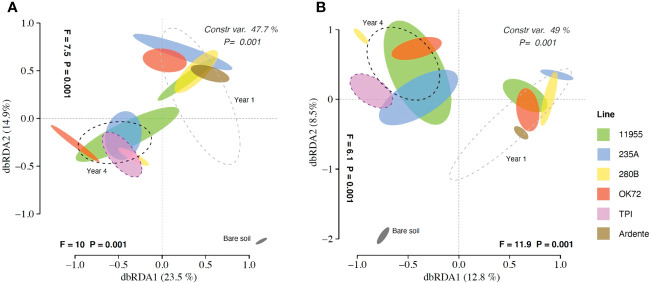
Distance-based redundancy analysis (dbRDA) plots showing shifts in bacterial **(A)** and fungal **(B)** community composition colonizing the rhizosphere of *Triticum durum* cv. Ardente, *Thinopyrum intermedium* (TPI) and four perennial wheat genotypes 235A, 280B, 11955, OK72 at the first and fourth year of growth.

Multivariate analyses on the total OTU matrices showed significant effects of plant genotypes and perenniality in shaping both bacterial and fungal communities. Indeed, dbRDA analysis reported a significant interaction “genotype” × “year” for bacteria (P = 0.019) and fungi (P = 0.02), with single factors “genotype” and “year” (P< 0.001) for both bacteria and fungi. The dbRDA model explains a percentage of the total variance of 47.7% (bacteria) (**Figure 1A**), and 49% (fungi) (**Figure 1B**). ADONIS showed that the rhizosphere and the bare soil microbial communities were significantly separated from each other (**Figures 1A, B**), indicating a significant impact of plants roots on selecting and shaping the rhizosphere microbial community. Moreover, for both bacteria and fungi, samples belonging to year 1 and year 4 were significantly different (p<0,005), together with the annual durum wheat and the parental species *Thinopyrum intermedium* respectively, suggesting a selection of the microbial community over the years. However, differences of microbial communities’ composition among wheat genotypes were not significant.

**Figure 2:**
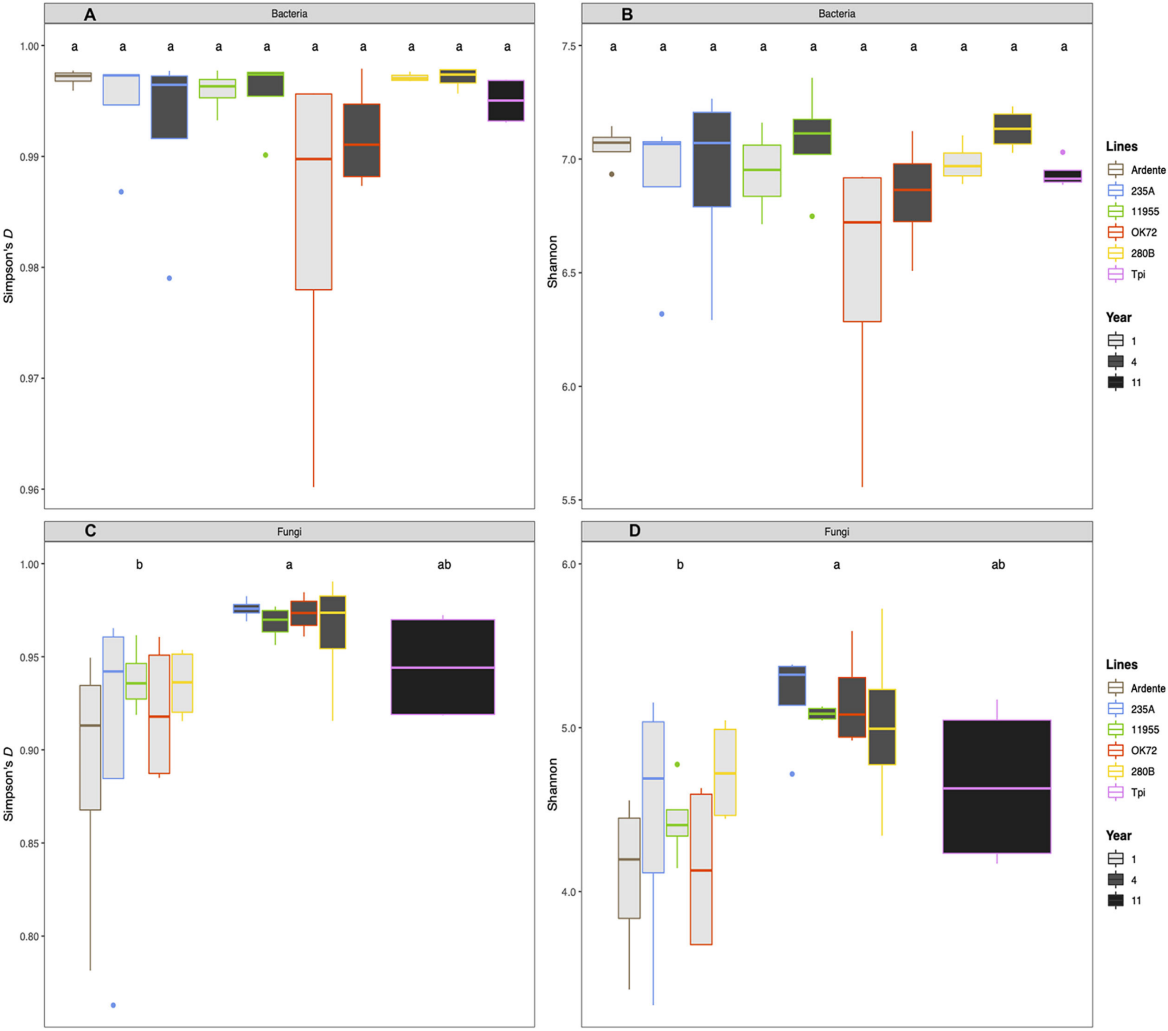
Mean values of Simpson’s *(D)* and index Shannon Wiever for bacteria **(A, B)** and fungi **(C, D)** as affected by time of plant residence on soil (1,4 and 11 years) as well as plant species/genotypes (*Triticum durum* cv. Ardente, *Thinopyrum intermedium* (Tpi) and four perennial wheat genotypes 235A, 280B, 11955, OK72). Different letters denote statistically different (Tukey’s test, P = 0.05) D-values among treatment.

Additionally, plant genotype had no significant effect on bacterial (**Figures 2A, B**) and fungal diversity (**Figures 2C, D**), while perenniality significantly increased the fungal evenness from the first to the fourth year of plant growth. Perenniality also increased the bacterial evenness even if not significantly. Phylogenetic analysis revealed a microbial composition and complexity with 20 phyla for bacteria (**Figure S1A**) and 6 phyla for fungi (**Figure S1B**). Among those, the most represented bacterial phyla were Proteobacteria (38%), followed by Bacteroidetes (12%), Actinobacteria (7%) and Verrucomicrobia (6%), while Ascomycota (33%), Basidiomycota (8%) and Zygomycota (6%) were the most abundant fungal phyla. A total of 458 bacterial genera and 331 fungal genera were also identified (see **Supplementary Datasets S1, S2**). About 25 bacterial genera had relative abundance >0.3% while only 10 fungal genera had relative abundance >0.03%. across all samples. Of these bacteria, the genera *Niabella*, *Flavisolibacter*, *Kaistobacter* and *Opitutus*, were more abundant in association with the roots of durum wheat and NPGs at the first year of growth. On the other hand, the rhizospheres of *Thinopyrum intermedium* and NPGs at the fourth year of growth were richer in the genera *Gemmata*, *Haliangium* and *Steroidobacter*. It is noteworthy that *Pseudomonas* genus (Phylum Proteobacteria) was highly abundant (more than 15% of the relative abundance) in association with the OK72 genotype both at the 1^st^ and 4^th^ year, (**SI Supplementary Mat Dataset S1**). At the 4^th^ year of cultivation, a greater abundance of fungi was detected, including the genera *Aspergillus*, *Chrysosporium* and *Fusarium*, among which plant pathogenic species occur (**SI Supplementary Mat Dataset S2**). Despite this, the higher fungal evenness at the 4^th^ year might contain the overgrowth of these fungi, as indicated by their low relative abundance (<1.5%). Moreover, the bacterial genus *Bacillus* and the fungal genus *Mortierella* to which belong beneficial species, were widely present across all samples.

### 3.2 Enzymatic activities and microbial biomass

**Figure 3:**
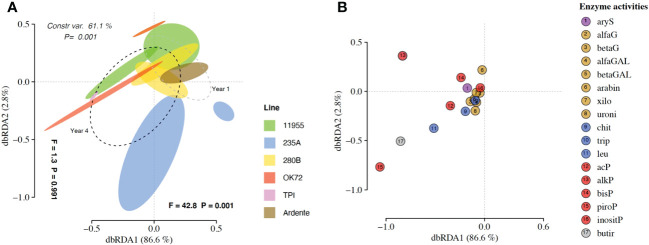
**(A)** Distance-based redundancy analysis (dbRDA) plot showing shifts in enzyme activities in the rhizosphere of *Triticum durum* cv. Ardente, *Thinopyrum intermedium* (TPI) and four perennial wheat genotypes 235A, 280B, 11955, OK72 at the first and fourth year of growth. Species scores corresponding to the dbRDA plots (coordinates for enzymes included in model) are reported in the scatter plots on the right **(B)**.

The dbRDA patterns of hydrolytic enzyme activity differed significantly among the treatment groups (P = 0.001) accounting for 61.1% of the total variance (**Figure 3A**). The factors “genotypes” and “years” caused separation along axes 1 (F = 1.3 and P = 0.991) accounting for 2.8% of the total variance and along axes 2 (F = 42.8 and P = 0.001) accounting for 86.6% of the total variance. ADONIS and dbRDA showed how enzyme profiles of *Thinopyrum intermedium*’ and durum wheat’s rhizospheres were clearly differentiated from each other as well as the enzyme profiles of OK72 and 235A genotypes at the 1^st^ and 4^th^ year of cultivation. On the contrary, the genotypes 11955 and 280B at the 1^st^ and 4^th^ year of cultivation were closer to each other. Species score plot (**Figure 3B**) showed that the higher activity (see **Supplementary Mat Dataset S5**) of alkaline (alkP) phosphomonoesterase, pyrophosphate-phosphodiesterase (piroP) and butyric esterase (butir) at the 4^th^ year of NPGs cultivation and associated with *Thinopyrum intermedium* compared to NPGs at the 1^st^ year and durum wheat, caused the horizontal differentiation in the dbRDA plot. This indicate that over the years production of P-acquiring enzymes (alkP and piroP) and enzymes involved in organic matter degradation (butir) increase under perennial wheats growth.

**Figure 4:**
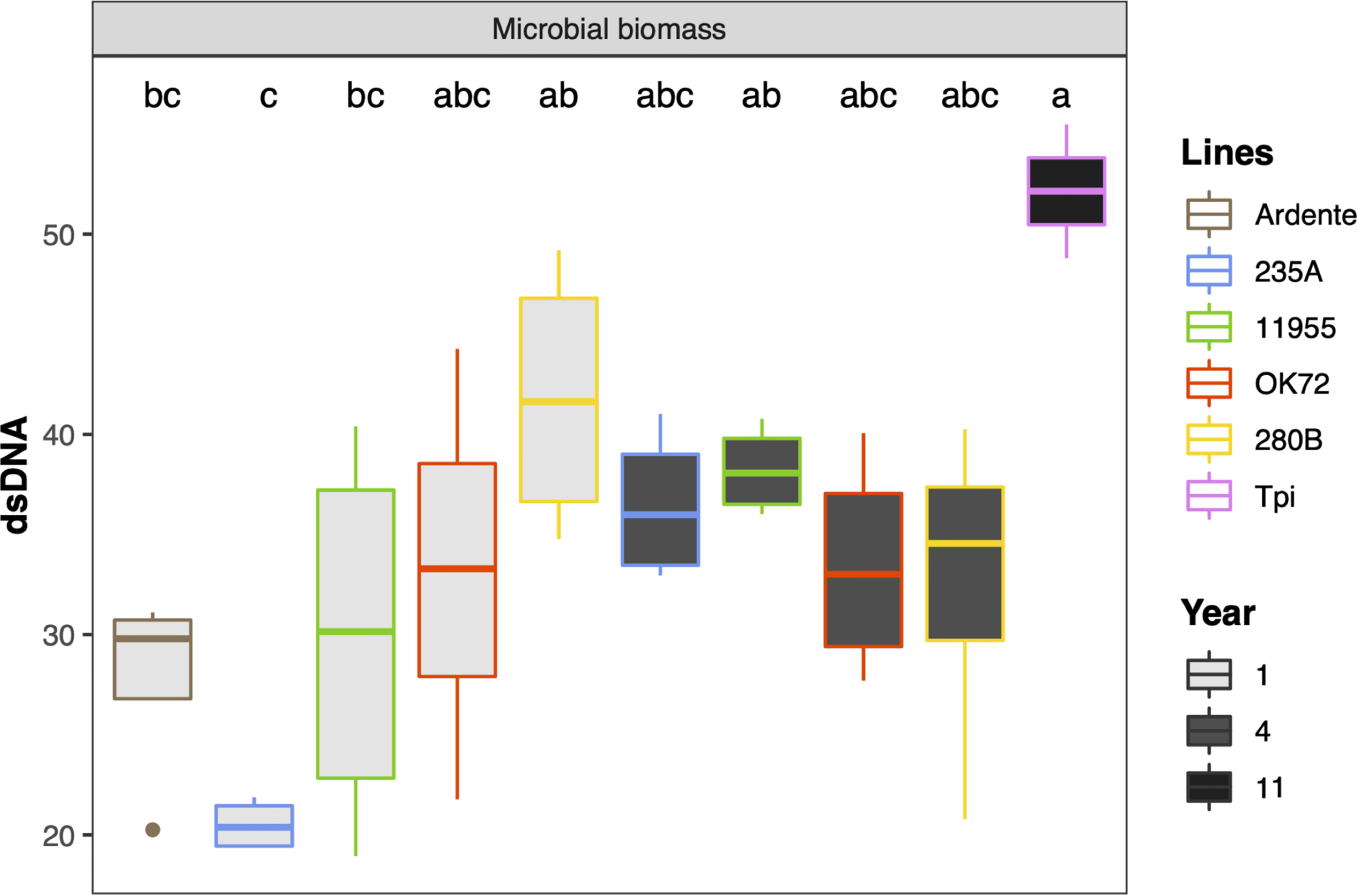
Mean values of microbial biomass as affected by time of plant residence on soil (1,4 and 11 years) and plant species/genotypes (*Triticum durum* cv. Ardente, *Thinopyrum intermedium* (Tpi) and four perennial wheat genotypes 235A, 280B, 11955, OK72). Different letters denote statistically different (Tukey’s test, P =0.05) D-values among treatment.

Univariate analysis on microbial biomass revealed that *Thinopyrum intermedium* had the highest microbial biomass, while at the 4^th^ year of cultivation it was similar among the NPGs (**Figure 4**). On the contrary, at the 1^st^ year of NPGs cultivation, the microbial biomass associated with genotype 280B was significantly higher than that associated to 235A, and not for the other genotypes. No statistical differences can be claimed between Ardente and NPGs, but only for the comparison Ardente/Thinopyrum.

### 3.3 Metabolomics

Following the GC-MS untargeted analysis of soil samples, 33 metabolites were annotated, as summarized in (**Supplementary Dataset 6**). Mono- and disaccharides (glucose, fructose, sucrose, trehalose) and sugar alcohol (mannitol/ribitol) were the most abundant biochemical classes detected. Polyalcohol, organic acids and nucleobases were also annotated but their intensities were lower compared to the other classes. These primary metabolites can accumulate in soils from leaves litter, root exudates, but are also produced by different microorganisms (Withers et al., 2020).

At first, the PCA was employed to explore the data. The first 3 PCs describes 58% of the total variance. As show in **Figure S2A**, a trend to separate samples according to the year of plant residence on soil is reported for the first PC. No clustering according to the genotypes was observed (**Figure S2B**).

**Figure 5:**
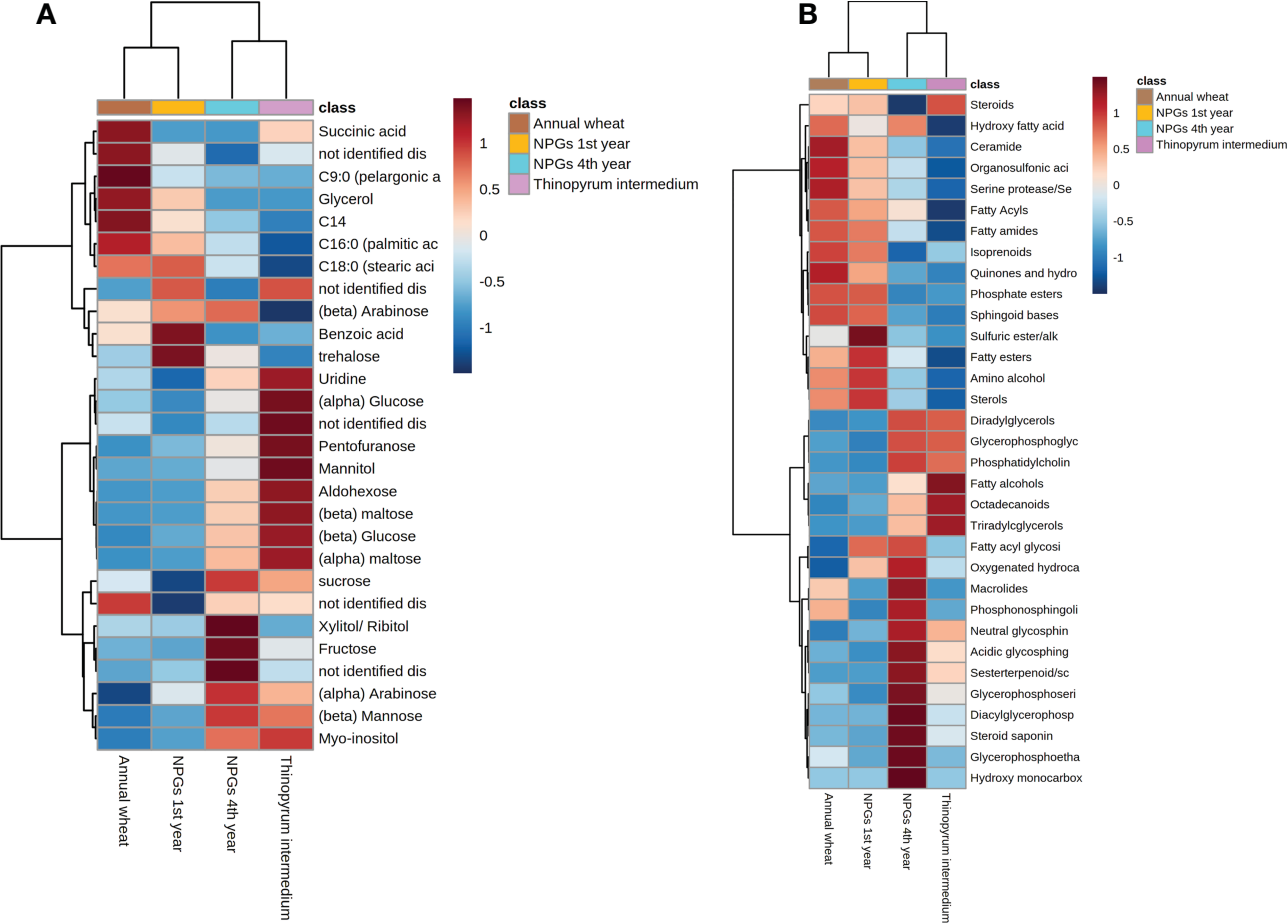
Hierarchical clustering analysis and heat map visualization obtained using annotated **(A)** metabolites and **(B)** lipids grouped in biochemical classes (distance: Euclidean; clustering algorithm: Ward).

Afterwards, supervised PLS-DA (**Figure S3**) was performed and displayed a clear differentiation between two main groups, i) samples belonging to NPGs’ rhizosphere collected at the 4^th^ year of growth with *Thinopyrum intermedium* (11 years), and ii) samples belonging to NPGs’ rhizosphere collected at the 1^st^ year of growth with *Triticum durum* cv. Ardente. The spread of the samples in the score plot as well as the low prediction ability of the PLS-DA model (*Q^2^* 0.179) is ascribable to the biological variability of the soil samples. Among the most significant metabolites responsible for this group separation, sugar alcohol, mono and disaccharides such as mannitol, glucose and maltose were accumulated in NPGs’ rhizosphere collected at the 4^th^ year and in *Thinopyrum intermedium* (11 years) (see heatmap in **Figures 5A**, **S4**). The opposite trend was observed for benzoic acid and glycerol, among other, whose intensities were significantly higher in soils collected at the 1^st^ year and decreased over time (4 and 11 years). Therefore, our results confirmed the importance of plant permanence on soil in shaping plant-soil polar metabolites.

### 3.4 Lipidomics

A total of 163 lipids grouped within 15 biochemical classes and 33 sub-classes were annotated (see **Supplementary Dataset S7**) among the statistically significant (FDR ANOVA p-value < 0.01) features. Triacylglycerol (TG) were the most abundant species followed by diacylglycerols (DG), fatty acids (FA), ceramides, sterols and prenol lipids. The mixing of various lipid sources in soil, as for sugars, makes difficult to determine if their origin is from plant or microorganism. Indeed, apart from glycolipid and sterols, the other lipid classes are common to most of the living organisms.

The sample clustering observed for lipids data (**Figure S5**) was in line with those observed for primary metabolites (**Figure S3**). TG and DG were accumulated in the rhizosphere collected at the 4^th^ year of growth and from *Thinopyrum intermedium*, compared to soils collected after 1 year of growth and *Triticum durum* cv (see heat map in **Figure 5B**). Same tendency was reported for glycerophospholipids, including glycerophosphocholine (PCs), glycerophosphoserine (PSs) and glycerophosphoethanolamines (PEs). On the other hands, opposite trend was observed for sphingoid bases, prenol lipids and fatty acids. Our results revealed that both storage (i.e., TG) and signalling (i.e., sphingolipids, fatty acids) lipids play a role in modulating the plant-microbial interaction, being statistically significant differentially accumulated in the rhizosphere collected at different years of plant permanence on soil.

## 4 Discussions

**Figure 6:**
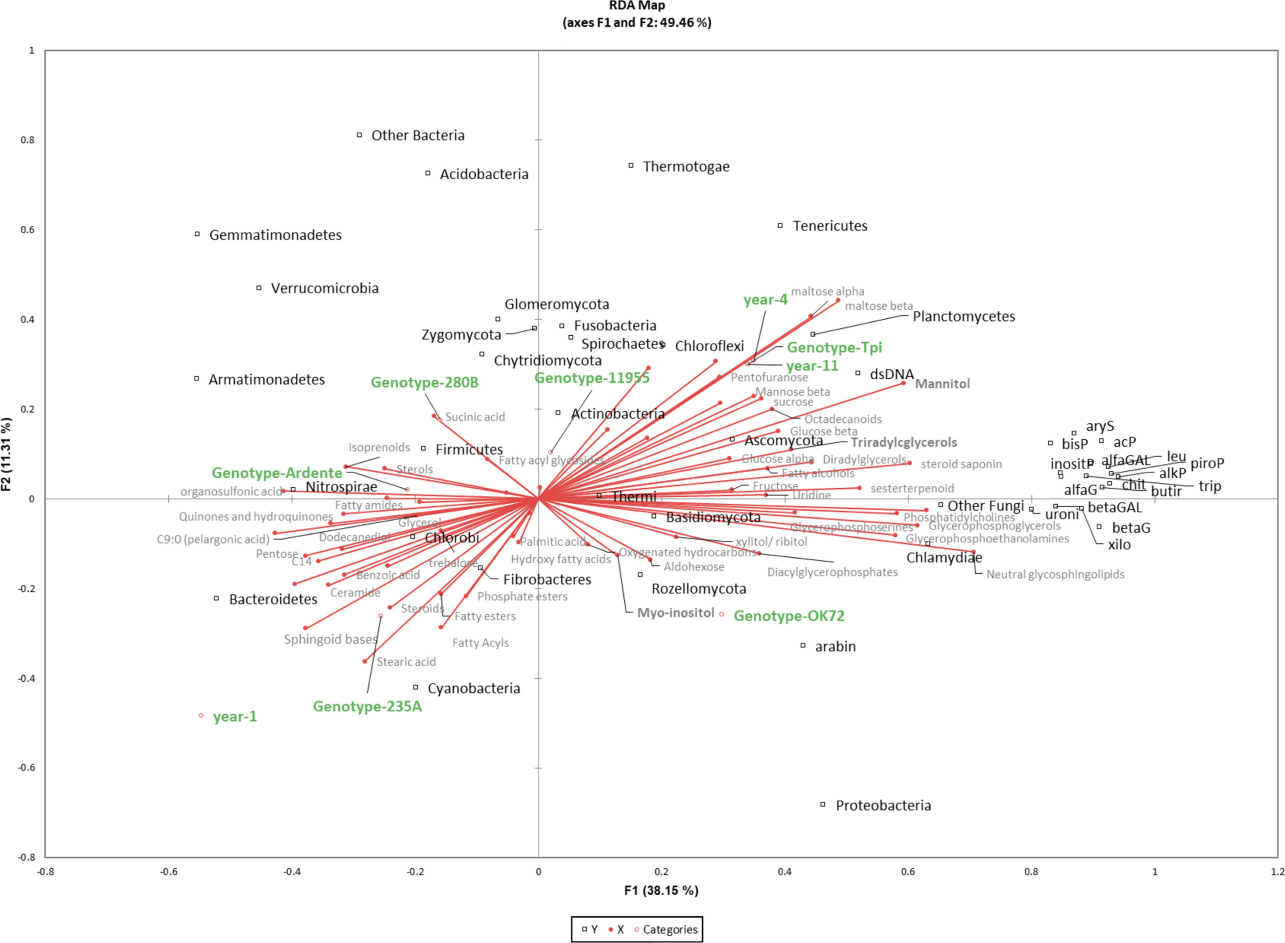
Redundancy analysis (RDA) of 16S rDNA, enzyme activity, metabolites and lipids in different species/genotypes (annual wheat-Ardente, NPGS- 280B, -235A, -11955, -OK72 and *T. intermedium*) and year of soil permanence (1_st_, 4_th_, 11_th_). The model accounted for 46% of the total inertia, with a global p-value < 0.0001. Squares (black) represent bacteria and fungi with their taxonomic affiliation; circles represent year and genotypes (green); metabolites and lipids are in grey palette.

A combination of -Omics techniques was applied to examine the rhizosphere environment of four new perennial wheat genotypes at the 1^st^ and 4^th^ year of cultivation in comparison to an annual durum wheat cultivar and the parental specie *Thinopyrum intermedium*. Our results showed that permanent soil cover and no-tillage, which characterize a perennial cropping system, have a predominant role in shaping perennial wheats rhizobiome and the rhizosphere environment. This is also confirmed by other studies where soil properties were the main factors structuring the rhizobiome (Schlatter et al., 2020; Simonin et al., 2020; Audu et al., 2022b), followed by crop management (Hargreaves and Hofmockel, 2014; Sprunger et al., 2019; Audu et al., 2022a), and crop genotypes (Brisson et al., 2019; Simonin et al., 2020; Ndour et al., 2021; Semchenko et al., 2021). In our study multivariate analysis on total bacterial and fungal OTUs, revealed a common rhizosphere microbial community composition between new perennial wheat genotypes at the 1^st^ year from sowing and the annual durum wheat. After four years of plant development, the composition of the rhizobiome mutated, resulting more similar to that one of the parental specie *Thinopyrum intermedium*, suggesting that the continuous development of perennial root system along the years affected the microbial community. Moreover, NPGs rhizobiome didn’t mutate after several years of plant establishment as shown by the similarity with *Thinopyrum intermedium* rhizosphere community, probably because with minor environmental disturbances the rhizosphere ecosystem saturates, becoming redundant (Yachi and Loreau, 1999). Despite the dominance of the perenniality effect, some differences in bacterial taxa were also observed by analyzing the 16S rDNA profiling between lines. In particular, *Pseudomonas* genus (phylum Proteobacteria) relative abundance was significantly higher in OK72 comparing to the other lines, regardless of the cultivation year (**Figures 6**, **S1A**). *Pseudomonas* strains are known to be able to control plant pathogens (Sah et al., 2021) and to respond well to the presence of root exudates by upregulating genes involved in the catabolism of myo-inositol (Mavrodi et al., 2021) which had been reported as an essential trait for the colonization of *Arabidopsis thaliana* roots (Cole et al., 2017). These enzymes release phosphorus (P) from myo-inositol P making it available for uptake by plants. *Pseudomonas* is also able to solubilize phosphorous from inorganic source, which is confirmed in our study by the positive correlation (p<0.0001) with myo-inositol and phosphorous acquiring enzymes (alkP, piroP and inositP) (See **Figure 6**, where is referenced the phylum, and **Supplementary Dataset S10**).

The presence of potential plant beneficial microbes involved in plant defense (Qiu et al., 2012; Liu et al., 2018; Lin et al., 2021) both at the 1^st^ and 4^th^ year of growth such as, *Flavisolibacter*, *Kaistobacter*, *Haliangium, Bacillus and Pseudomonas*, might be of particular interest since these perennial wheat derivatives never showed powdery mildew and rusts (*Puccinia* spp.) disease symptoms during our experiment as well as in other field experiments (Pogna et al., 2014). This is noteworthy since perennial grains are generally considered susceptible to diseases (Cox et al., 2005). Indeed, typical cultural practices effective at reducing soil- and residue-borne pathogens, such as annual crop rotations, delayed fall planting, and tillage, are not feasible in perennial systems. Moreover, the higher fungal evenness detected on NPGs after four years might be also involved in the phenomenon of the general soil-borne disease suppressiveness, controlling the spread of pathogenic (Mazzola, 2002). Indeed, when many different species are present, they can fulfil a variety of different ecological niches within a given ecosystem, thus competing with potential plant pathogen. This result is confirmed also by the lipid analysis. Indeed, phytosphingosines are significantly accumulated in the rhizosphere after one and four years most likely as root exudates. These compounds have been recently demonstrated to be produced by plants following plant-pathogen interaction as a resistance strategy (Glenz et al., 2022).

However, the prolonged absence of significant soil disturbances, as in the case of *Thinopyrum intermedium* which is resident in the same soil for 11 years, led to fungal evenness reduction, which could jeopardize the resistance to biotic stresses. Indeed, according to the insurance hypothesis, biodiversity insures ecosystems against declines in their functioning because many species provide greater guarantees that some will maintain functioning even if others fail (Yachi and Loreau, 1999). Moreover, *Thinopyrum intermedium* reported the highest microbial biomass due to the reduced environmental disturbance and the constant supply of carbon from above- and belowground plant biomass as well as root exudates (Allison et al., 2005; Culman et al., 2010; Liang et al., 2012; Xia et al., 2019). Further, significant differences among plant genotypes (280B and 235A) at the first year of growth were detected for microbial biomass. Based on a previous study, these differences might be caused by a dissimilar development strategy of the roots’ apparatus of the lines which showed variability in agronomic and eco-physiological performance in two subsequent years of cultivation (Baronti et al., 2022). However, the progressive reduction of differences in microbial biomass between genotypes after four years, might be the consequence of the vertical root development and the continuous deposition of aboveground plant litter on soil. Moreover, plant litter accumulation, root turnover and the continuous production of root exudates for many years had boosted the activity of all the enzymes considered in this study and significantly the enzymes involved in organic matter decomposition (butir) (Hargreaves and Hofmockel, 2014) and of phosphorous acquiring enzymes (alkP and piroP) (See **Figure 6**). Butyrate esterase is generally related to microbial biomass content (Kähkönen et al., 1999; Trivedi et al., 2016), while changes in microbial community structure and diversity over the years are not reflected, with the same extent, to an improvement of the functional diversity. This indicate that decomposition of organic matter is not related to the microbial diversity since most microorganisms can carry out this function (Nannipieri et al., 2003). Moreover, the increase of rhizodeposit quantity, but with small changes in their composition is not sufficient to change soil functionality, because there is no need to change enzyme pattern when the substrate is very similar.

Furthermore, perennial wheats root development along the years modifies the chemical properties of the rhizosphere environment. In our study, the abundance of rhizosphere metabolites is consistent with other research (Liu et al., 2020; Iannucci et al., 2021), where the most abundant are usually sugars, followed by polyalcohols, organic acids, and amino acids. They are involved in a variety of functions including the modulation of nutrient availabilities, mainly by means of soil acidification (Carvalhais et al., 2011), and in plant–microbe interactions (Bais et al., 2006; Liu et al., 2020; Bi et al., 2021). Indeed, sugars, independent of origins (roots, bacteria, fungi) improve soil structure by affecting aggregates formation and water retention, contributing to C stabilization and creating a favorable environment for both root development and microbial growth (Gunina and Kuzyakov, 2015). However, sugars are the energy source for all the living organisms, making challenging the identification of their origin in soil. Indeed, it is estimated that only 20% of the carbohydrates in soils are exudated from plant, while the remaining 80% originates from the secondary source, which are microorganisms and their residue (Gunina and Kuzyakov, 2015). The higher content of hexose, including glucose, in the rhizosphere collected at the 11^th^ year may be the result of the repetitive and thus, cumulative accumulation both as root exudate (originated from cellulose decomposition) and microorganism synthesis. Indeed, its trend 11^th^ year > 4^th^ year >1^st^ year is consistent with the continuous development of perennial root system along the years, which, in turn, affects the microbial biomass as highlighted by the RDA analysis in **Figure 6**. Moreover, we observed a positive correlation (p<0.01) between glucose and betaG enzyme activity which is further correlated with Proteobacteria and Actinobacteria (p<0.01) (see **Supplementary Dataset S10**). These are the main bacterial phyla, while Ascomycota is the only dominant fungal phylum that exhibit high betaG enzyme activity, as recently reported (Zeng et al., 2022).

Together with glucose, maltose, and mannitol, significantly accumulated in the rhizosphere along the years (see **Figures 6**, **S4**). These metabolites are commonly associated with osmotic regulation (Brown et al., 2021), suggesting that along the years both plants and microorganisms are led to maintain a favorable environment for their growth. Interestingly, mannitol is synthesized in numerous plant species, but not in common wheat (*Triticum aestivum*) (Abebe et al., 2003). Therefore, its accumulation has to be mediated by fungal and bacteria metabolism (Patel and Williamson, 2016). We observed the same trend for TGs, that can be accumulated in soil mainly as root exudates. They are a major energy deposit for most of eukaryotic organisms, including fungi, yeast, plants and animals but occurrence in bacteria is limited to the actinomycetes taxa (Alvarez and Steinbchel, 2002). Their accumulation in the rhizosphere collected at the 4^th^ and 11^th^ year may result from i) a higher excretion from the continuous development of perennial root system, as well as ii) from a lower degradation rate occurring in the soil but also iii) due to the higher presence of actinomycetes in the rhizosphere (positive correlation p < 0.001). Indeed, TGs in soil undergo β-oxidation with the formation of FAs (Hita et al., 1996), which in our study were found to be accumulated in the annual wheat and NPGs after one years. Fatty acids are essential molecules that play crucial roles in plant-plant, plant-microbe and plant-environment interactions (Macabuhay et al., 2022). Furthermore, some studies have revealed that fatty acids and their derivatives directly inhibit the growth of plant pathogens within the rhizosphere, and improve the surrounding environment of plant rhizosphere to reduce the occurrence of crop diseases and promote crop growth (Upchurch, 2008; Raffaele et al., 2009). In our study, wheat perenniality has led to an increase in abundance of complex lipids, glycerolipids and the glycerophospholipids. These are involved in many regulatory processes such as cell signaling and intracellular trafficking (Macabuhay et al., 2022). In addition, the higher amount of glycerophospholipids and glycerolipids in the microbial plasma membrane can be an adaptive trait for the well-established and selected microbial community of NPGs rhizosphere after 4 years. In particular, phosphatidil ethanolammine showed to be significantly more abundant in the 4^th^ year NPGs respect to the annual wheat line. Moreover, the increase in glycerophospholipids reduces microbes membrane permeability and increases resistance against antimicrobial compounds allowing the establishment of coexistence between different microbial species rather than their eradication (Molina-Santiago et al., 2021). Furthermore, they are released as exudates of plant roots and their role in directly influencing growth of plant interacting microorganisms has been only recently investigated (Glenz et al., 2022; Zeng and Yao, 2022). In addition, the increased level of hydrophobic components of organic matter can enhance soil aggregate stability and C stabilization (Goh, 2004). However, the study of the soil lipidome is a new frontier of research and very few studies were conducted so far (Tinoco et al., 2018; Wilson et al., 2021). Even though the role of lipid in rhizodeposition and plant-microbe signaling (Bi et al., 2021) as well as microbes-to-plant signaling (Macabuhay et al., 2022) is undoubtedly relevant, the interpretation of these data remain challenging due to the lack of knowledge.

**Figure 7:**
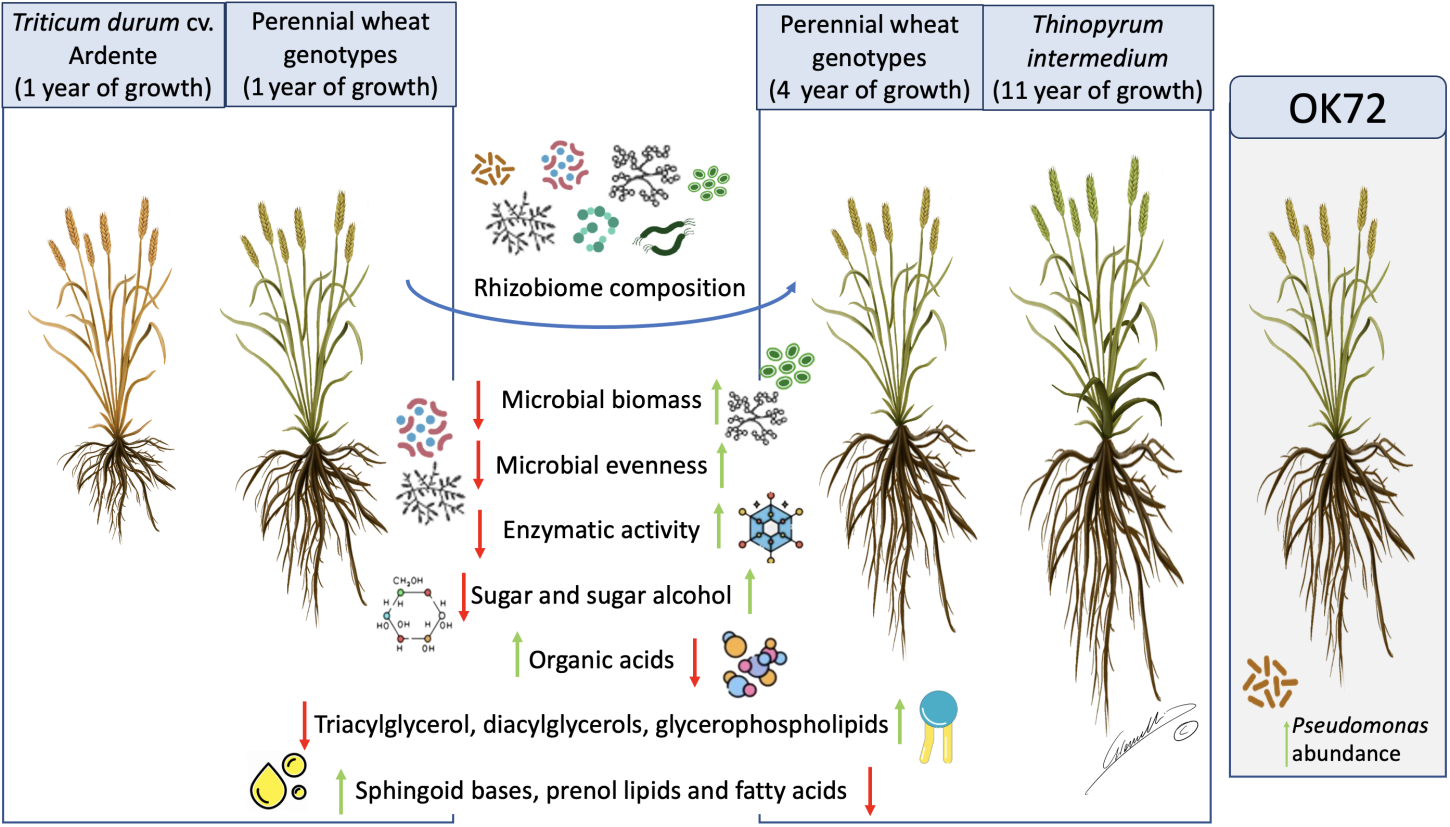
Schematic representation of the results obtained in this work. Green arrows represent increasing in abundance while red arrows represent decreasing in abundance in the different samples analyzed. OK72 line was highlighted as promising genotype for selection of new perennial wheat lines. Image by Gianluigi Giannelli (University of Parma).

In conclusion, in this study, we demonstrate that the long permanence of perennial wheat lines on soil (see **Figure 7**) remarkably modifies the quality and quantity of root exudates, shaping the rhizosphere microbial community composition and favoring the growth of microorganisms as indicated by their higher biomass and activity. Moreover, these modifications point to the existence of a less stressful environment which favor the plant-microbiome crosstalk, thus improving plant resilience to biotic and abiotic stresses. Despite the dominance of the perenniality effect, OK72 distinguished from the other lines not only for its better agronomic features and nutritional quality, as previously shown (Gazza et al., 2016; Baronti et al., 2022) but also for the increase of potential beneficial microorganisms such as *Pseudomonas* spp., thus resulting a suitable candidate for the study and selection of new perennial wheat lines (**Figure 7**).

## Data availability statement

The data of 16S rDNA and ITS sequences presented in the study are deposited in the NCBI database at the following link https://www.ncbi.nlm.nih.gov/sra/PRJNA826315.

## Author contributions

GV, MB, and LR planned and designed the research, MB performed the experiments, FF performed soil enzymatic activities, LG conducted fieldwork, MB, GV, AF, LR, MC, VL analysed data. GV, LR supervise the work. GG funded the research, MB, GV, LR wrote the manuscript, all the authors revise the manuscript. All authors contributed to the article and approved the submitted version.
