# Supplementary material for: Perenniality, more than genotypes, shapes biological and chemical rhizosphere composition of perennial wheat lines
Source: Front Plant Sci. 2023 May 8;14:1172857. doi: 10.3389/fpls.2023.1172857 (PMC10200949; doi:10.3389/fpls.2023.1172857)
Supplement: Supplementary file 8 [file Table_8.docx]

Data set S8

Num samples: 44

Num observations: 21,996

Total count: 2,790,088

Table density (fraction of non-zero values): 0.264

Counts/sample summary:

Min: 29,614.000

Max: 210,928.000

Median: 61,700.000

Mean: 63,411.091

Std. dev.: 26,998.702

Sample Metadata Categories: GenotypeSoilType; Description; GenotypeYear; SoilType; SoilGenotypeYear; SoilTypeYear; Year; Genotypes

Observation Metadata Categories: taxonomy

Counts/sample detail:

ID2205-V3V4-7-7-P01-G01: 29,614.000

ID2205-V3V4-21-20-P01-E03: 34,903.000

ID2205-V3V4-8-8-P01-H01: 36,419.000

ID2205-V3V4-20-19B-P01-D03: 37,457.000

ID2205-V3V4-6-6-P01-F01: 41,813.000

ID2205-V3V4-16-16-P01-H02: 42,920.000

ID2205-V3V4-34-33-P01-B05: 43,069.000

ID2205-V3V4-5-5-P01-E01: 44,126.000

ID2205-V3V4-33-32-P01-A05: 44,166.000

ID2205-V3V4-14-14-P01-F02: 47,480.000

ID2205-V3V4-4-4-P01-D01: 48,056.000

ID2205-V3V4-10-10-P01-B02: 48,063.000

ID2205-V3V4-19-19A-P01-C03: 49,620.000

ID2205-V3V4-28-27-P01-D04: 50,223.000

ID2205-V3V4-1-1-P01-A01: 51,180.000

ID2205-V3V4-26-25-P01-B04: 51,438.000

ID2205-V3V4-3-3-P01-C01: 52,045.000

ID2205-V3V4-11-11-P01-C02: 54,590.000

ID2205-V3V4-43-41A-P01-C06: 54,778.000

ID2205-V3V4-17-17-P01-A03: 57,384.000

ID2205-V3V4-22-21A-P01-F03: 60,113.000

ID2205-V3V4-41-40A-P01-A06: 61,436.000

ID2205-V3V4-32-31B-P01-H04: 61,964.000

ID2205-V3V4-44-41B-P01-D06: 62,802.000

ID2205-V3V4-30-29-P01-F04: 63,596.000

ID2205-V3V4-40-39-P01-H05: 65,545.000

ID2205-V3V4-36-35-P01-D05: 65,778.000

ID2205-V3V4-31-31A-P01-G04: 67,045.000

ID2205-V3V4-15-15-P01-G02: 69,008.000

ID2205-V3V4-25-24-P01-A04: 70,854.000

ID2205-V3V4-35-34-P01-C05: 71,028.000

ID2205-V3V4-42-40B-P01-B06: 72,420.000

ID2205-V3V4-12-12-P01-D02: 72,422.000

ID2205-V3V4-29-28-P01-E04: 72,855.000

ID2205-V3V4-13-13-P01-E02: 73,257.000

ID2205-V3V4-39-38-P01-G05: 73,983.000

ID2205-V3V4-37-36-P01-E05: 76,257.000

ID2205-V3V4-2-2-P01-B01: 79,078.000

ID2205-V3V4-27-26-P01-C04: 80,724.000

ID2205-V3V4-38-37-P01-F05: 83,490.000

ID2205-V3V4-18-18-P01-B03: 83,882.000

ID2205-V3V4-9-9-P01-A02: 85,510.000

ID2205-V3V4-23-21B-P01-G03: 86,769.000

ID2205-V3V4-24-23-P01-H03: 210,928.000
