# Supplementary material for: Perenniality, more than genotypes, shapes biological and chemical rhizosphere composition of perennial wheat lines
Source: Front Plant Sci. 2023 May 8;14:1172857. doi: 10.3389/fpls.2023.1172857 (PMC10200949; doi:10.3389/fpls.2023.1172857)
Supplement: Supplementary file 9 [file Table_9.docx]

Num samples: 44

Num observations: 17,912

Total count: 2,377,941

Table density (fraction of non-zero values): 0.104

Counts/sample summary:

Min: 18,298.000

Max: 447,040.000

Median: 45,555.000

Mean: 54,044.114

Std. dev.: 62,155.334

Sample Metadata Categories: GenotypeSoilType; Description; GenotypeYear; SoilType; SoilGenotypeYear; SoilTypeYear; Year; Genotypes

Observation Metadata Categories: taxonomy

Counts/sample detail:

ID2323-ITS-1-1-P01-A01: 18,298.000

ID2323-ITS-20-19B-P01-D03: 19,253.000

ID2323-ITS-14-14-P01-F02: 22,786.000

ID2323-ITS-13-13-P01-E02: 24,376.000

ID2323-ITS-41-40A-P01-A06: 24,816.000

ID2323-ITS-16-16-P01-H02: 26,036.000

ID2323-ITS-44-41B-P01-D06: 26,383.000

ID2323-ITS-29-28-P01-E04: 27,763.000

ID2323-ITS-15-15-P01-G02: 28,910.000

ID2323-ITS-8-8-P01-H01: 29,959.000

ID2323-ITS-12-12-P01-D02: 31,000.000

ID2323-ITS-27-26-P01-C04: 31,330.000

ID2323-ITS-30-29-P01-F04: 32,819.000

ID2323-ITS-7-7-P01-G01: 35,931.000

ID2323-ITS-10-10-P01-B02: 36,263.000

ID2323-ITS-24-23-P01-H03: 36,311.000

ID2323-ITS-40-39-P01-H05: 37,287.000

ID2323-ITS-25-24-P01-A04: 39,946.000

ID2323-ITS-5-5-P01-E01: 40,438.000

ID2323-ITS-9-9-P01-A02: 40,690.000

ID2323-ITS-32-31B-P01-H04: 41,101.000

ID2323-ITS-2-2-P01-B01: 45,485.000

ID2323-ITS-19-19A-P01-C03: 45,625.000

ID2323-ITS-36-35-P01-D05: 46,170.000

ID2323-ITS-11-11-P01-C02: 46,261.000

ID2323-ITS-3-3-P01-C01: 47,298.000

ID2323-ITS-42-40B-P01-B06: 48,771.000

ID2323-ITS-37-36-P01-E05: 49,405.000

ID2323-ITS-6-6-P01-F01: 49,426.000

ID2323-ITS-39-38-P01-G05: 49,615.000

ID2323-ITS-22-21A-P01-F03: 50,000.000

ID2323-ITS-43-41A-P01-C06: 50,921.000

ID2323-ITS-28-27-P01-D04: 52,569.000

ID2323-ITS-35-34-P01-C05: 58,146.000

ID2323-ITS-38-37-P01-F05: 64,176.000

ID2323-ITS-33-32-P01-A05: 64,554.000

ID2323-ITS-23-21B-P01-G03: 65,396.000

ID2323-ITS-34-33-P01-B05: 66,100.000

ID2323-ITS-26-25-P01-B04: 69,742.000

ID2323-ITS-31-31A-P01-G04: 73,760.000

ID2323-ITS-21-20-P01-E03: 74,241.000

ID2323-ITS-17-17-P01-A03: 78,177.000

ID2323-ITS-4-4-P01-D01: 83,367.000

ID2323-ITS-18-18-P01-B03: 447,040.000
