# Supplementary material for: Perenniality, more than genotypes, shapes biological and chemical rhizosphere composition of perennial wheat lines
Source: Front Plant Sci. 2023 May 8;14:1172857. doi: 10.3389/fpls.2023.1172857 (PMC10200949; doi:10.3389/fpls.2023.1172857)
Supplement: Supplementary file 11 [file DataSheet_1.docx]

Perenniality, more than genotypes, shapes biological and chemical rhizosphere composition of perennial wheat lines

Marta Bertola^a,1^, Laura Righetti^a,b,c*^, Laura Gazza^d^, Andrea Ferrarini^e^, Flavio Fornasier^f^, Martina Cirlini^a^, Veronica Lolli^a^, Gianni Galaverna^a^, Giovanna Visioli^g,*^

**Supplementary Information**

The following Supporting Information is available for this article:

**Fig. S1** Taxonomic distribution based on 16S rDNA and ITS rDNA reads annotated lipids grouped into biochemical classes

**Fig. S2** Unsupervised principal components analysis (PCA) models built from GC-MS annotated metabolites

**Fig. S3** PLS-DA models built from GC-MS annotated metabolites

**Fig. S4** Metabolites and lipids changes in the rhizosphere

**Fig. S5** Multivariate modelling built from annotated lipids grouped into biochemical classes

**Dataset S1** contains the relative abundances of bacteria phylum, classes, order, family, and genera.

**Dataset S2** contains the relative abundances of fungal phylum, classes, order, family, and genera.

**Dataset S3** contains the OTUs abundances obtained by 16S rRNA NGS sequencing which were normalized to 10000 reads and used for α-diversity and β-diversity estimations.

**Dataset S4** contains the OTUs abundances obtained by ITS NGS sequencing which were normalized to 10000 reads and used for α-diversity and β-diversity estimations.

**Dataset S5** contains enzyme activity analysis (EAA) results on 17 hydrolytic enzymes involved in the principal nutrient cycles and results relative to microbial biomass (dsDNA).

**Dataset S6** contains the GCMS results in relative abundances.

**Dataset S7** contains raw and annotated lipids**.**

**Dataset S8** is a summary table of utilized reads per sample (after any overlap or filtering routine).

**Dataset S9** is a summary table of utilized reads per sample (after any overlap or filtering routine).

**Dataset S10** is the correlation matrix among bacterial phylum, fungal phylum, enzymatic activity, primary metabolites and lipids.

**Figures**

A


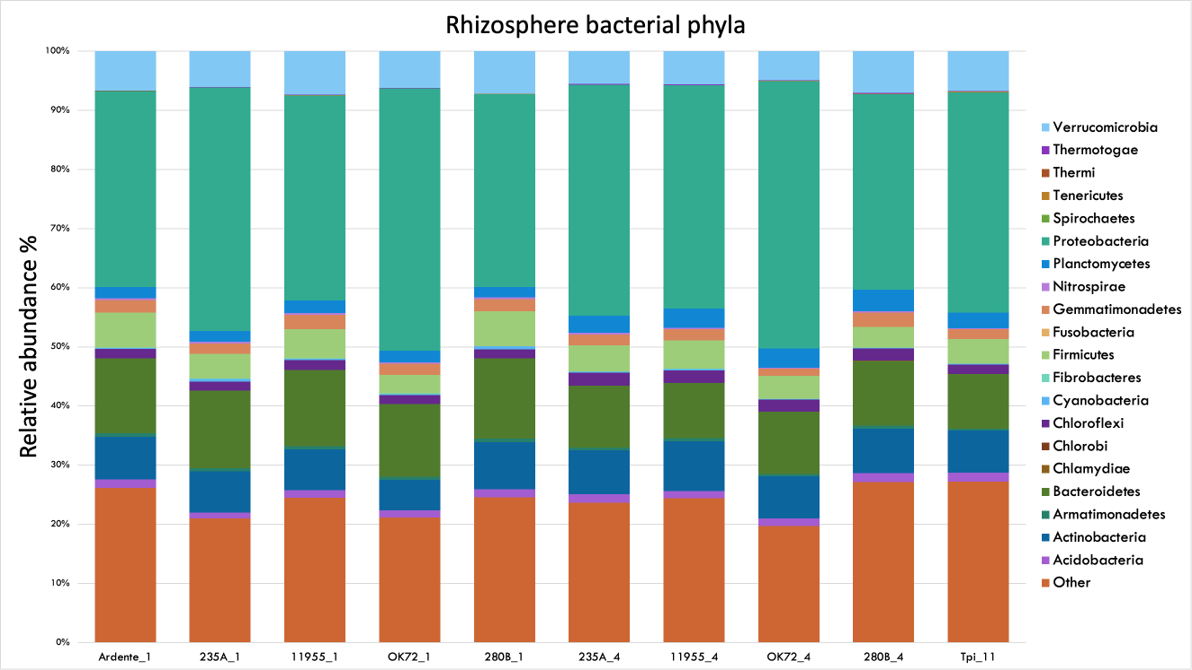


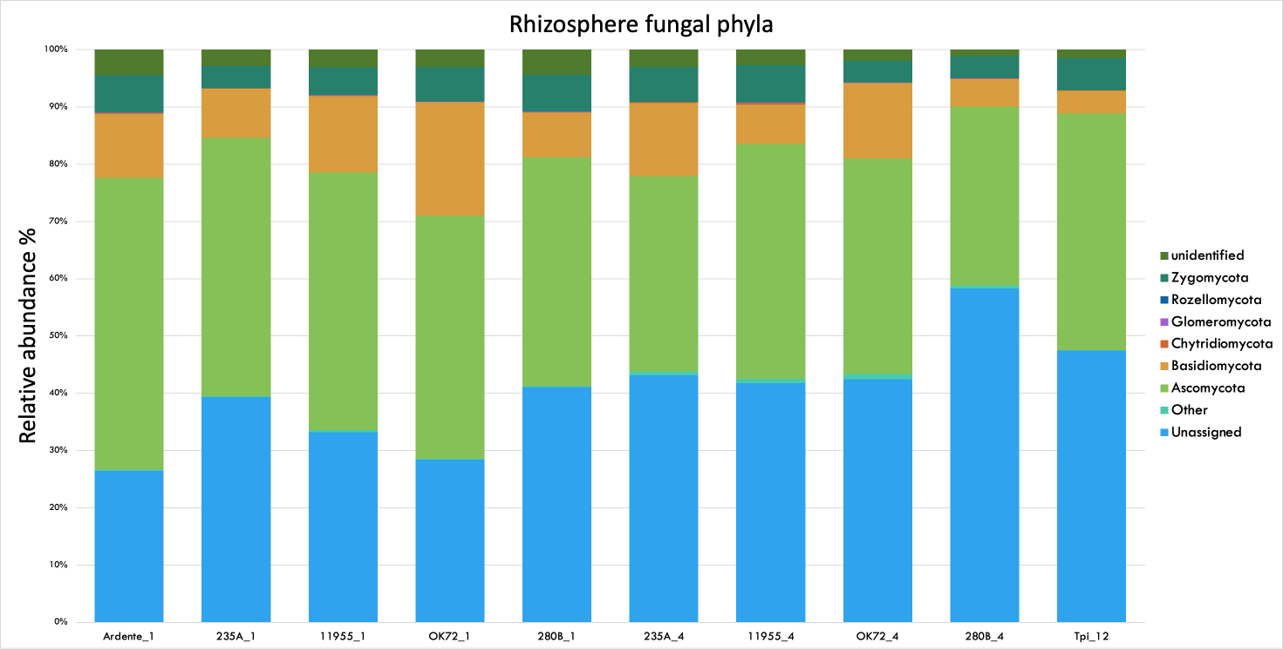


B

**Fig. S1** Taxonomic distribution at the phylum level based on 16S rDNA read (A) and ITS rDNA read (B) of bacteria and fungi associated with Triticum durum cv. Ardente, Thinopyrum intermedium and four perennial wheat genotypes 235A, 280B, 11955, OK72 at the first and fourth year of growth.

**
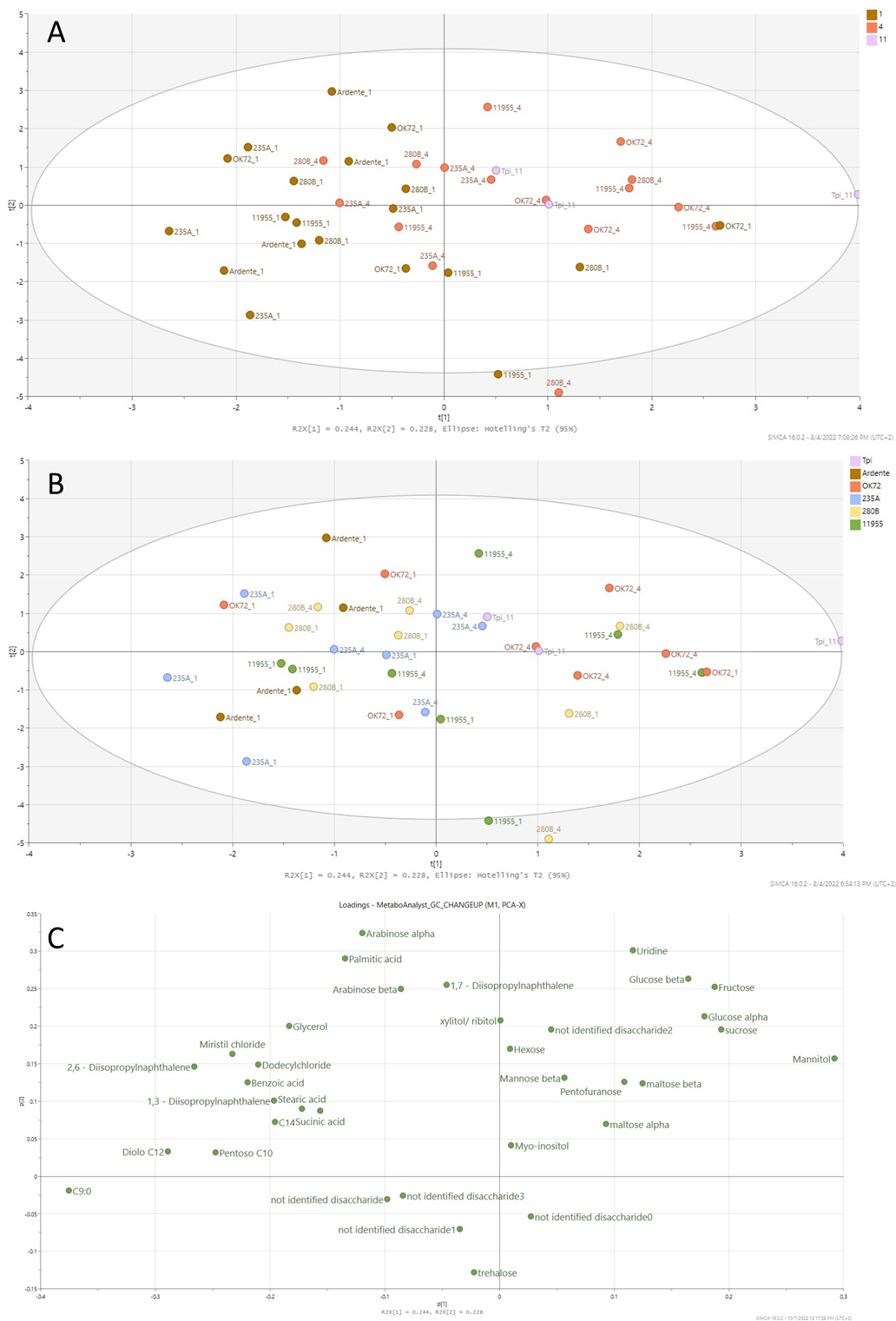
**

**Fig. S2** Unsupervised principal components analysis (PCA) models built from GC-MS annotated metabolites and colored according to (A) years of plant permanence on soil and (B) genotypes. (C) PCA loading plot highlighting the most discriminative metabolites


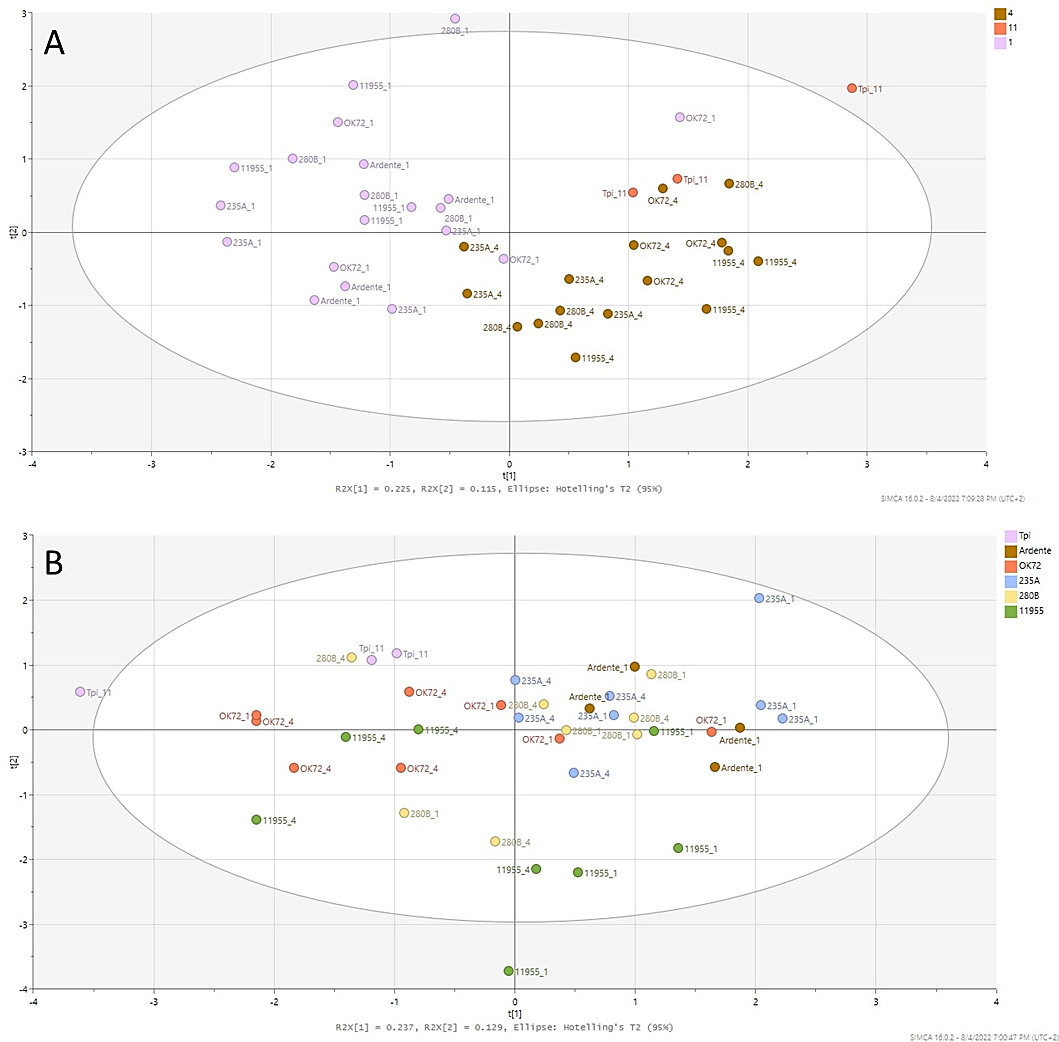
**Fig. S3** PLS-DA models built from GC-MS annotated metabolites and classified according to (A) years of plant permanence on soil and (B) genotypes.


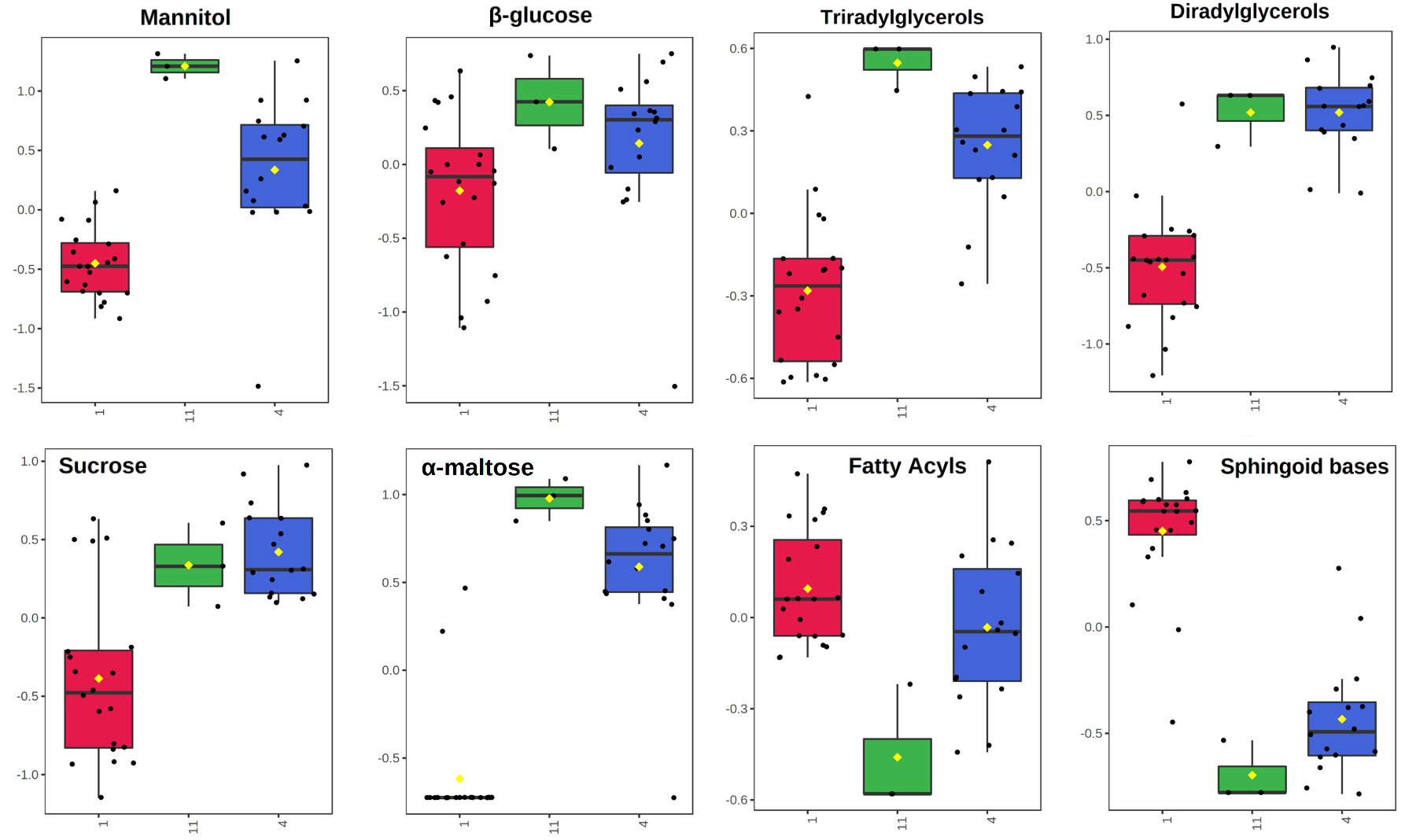
**Fig. S4** Metabolites and lipids changes in the rhizosphere depending on the years (1, 4 or 11) of plant permanence on soil.


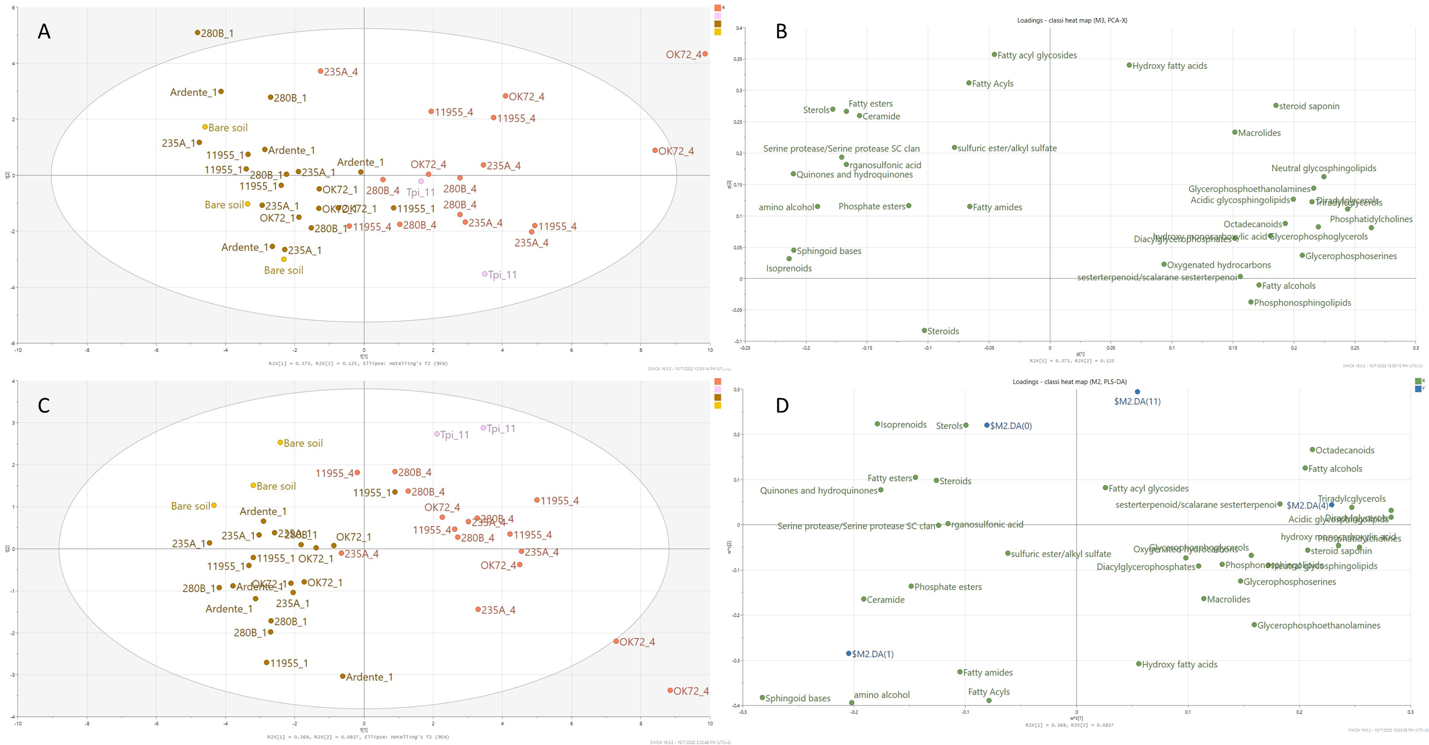


**Fig. S5** Multivariate modelling built from annotated lipids grouped into biochemical classes and colored according to years of plant permanence on soil: (A) PCA and the corresponding (B) loading plot, (C) PLS-DA and (D) its loading plot highlighting the most discriminative lipid classes.
